# Chicago Medical Society

**Published:** 1867-02

**Authors:** 


### PROCEEDINGS OF MEDICAL SOCIETIES.

### CHICAGO MEDICAL SOCIETY,

### SINGULAR PARASITE.

Dr. Ernst Schmidt exhibited a specimen of a very unusual parasitic worm, with the following history:—An American girl, 10 years of age, weakly, and of scrofulous diathesis, had suffered, for some time, from capricious appetite and pain in the upper portion of the abdomen. Two weeks ago she complained of pain on the left shoulder; at the seat of pain there soon appeared a slight elevation, tender to the touch, slightly red, from which, in a short time, a small worm was observed, endeavoring to escape. If disturbed, the worm withdrew its head beneath the skin; by gentle pressure, however, it was forced through the small opening it had made. A similar swelling appeared behind the left ear, from which another worm made its escape. Thus far, eight elevations have appeared in different portions of the body, from which five worms have been secured. Some of these elevations were neither red nor very tender on pressure. Palpation gave the sensation of a foreign body under the skin. The efforts of the worms to pass through the skin caused no pain nor hemorrhage. The small opening closed readily, and the elevation soon subsided.

The child, during this time, was nervous and irritable, the pulse being, at times, as low as 40 per minute. The application of mercurial ointment caused the elevations to disappear. In all cases where this was applied, the worms seemed to pass upward from their original position towards the head. The child was not in the habit of eating either raw meat or vegetables. There were no indications of other parasitic worms in the child or other members of the family.

The worms were about five-eighths of an inch in length, quite transparent, and divided into eight sections, with an alimentary canal running nearly straight from the anterior to the posterior extremity. The mouth, as examined with the microscope, presented no exidences of apparatus for suction or boring.

Dr. Schmidt will give, at some future time, a further history of the case, with a minute description of this rare parasite.

### LARYNGEAL TUMOR.

Dr. Ross exhibited the larynx, of a female patient 42 years of age, who died at the County Hospital, under the following circumstances:—She had enjoyed excellent health until last May, when she contracted a "severe cold" from exposure. From this time, she suffered from loss of voice and inflammation of the fauces, which symptoms were partially revived by applications to the mucous membrane and to the external parts of the throat by her attending physician. The respiration, on the contrary, became more and more impeded, till, finally, she was obliged to seek relief at the hospital, January 1st, 1867. At this time, she appeared in good general health. Both expiration and respiration were labored and accompanied by a loud, wheezing sound. The countenance was anxious; the pulse 90 per minute, full and strong; deglutition was difficult; there was a troublesome cough, with expectoration of thick mucus, which was at times bloody. The patient had never suffered from syphilis or from other chronic disease. The laryngoscope revealed a tumor, of considerable size, situated, apparently, between the anterior portion of the vocal cords. The glottis was nearly closed; the epiglottis partially destroyed. No ulceration of the mucous membrane was discovered.

Laryngotomy was performed by Dr. Bogue, with temporary relief, however, for only three days. Death occurred Jan. 14.

The *post mortem* examination, as shown by this specimen, revealed the large tumor, attached to the larynx just above the anterior part of the vocal cords. Below the vocal cords was a small but very deep ulceration, resembling the malignant ulcer not unfrequently observed in the tonsils.

### RARE DISEASE OF THE TRICUSPID VALVES.

Dr. Ross exhibited the heart, removed from a girl, 13 years of age, who had suffered from rheumatism at different times for the past six years. She entered the hospital with hypertrophy and valvular disease of the heart, with pleuritic and pericardiac effusion, and general anasarca. The heart weighed thirteen and one-half ounces. The pericardium contained sixteen and the pleural cavity thirty-two ounces of fluid. The aortic, semilunar, and mitral valves were hard and much thickened, closing very imperfectly. The most remarkable abnormal change was a very extensive thickening and contraction of the curtains of the tricuspid valves, presenting the appearance of a white tumor with a button-hole slit through it.

Dr. Ross also exhibited two hypertrophied hearts, without valvular lesion, weighing, respectively, eighteen and twenty-three ounces. The specimens were removed from patients suffering from Bright's disease.

Dr. Hildreth read the history of a case of perforating ulcer of the cornea, resulting from purulent conjunctivitis. Although there was continued evacuation of the aqueous humor through the opening in the cornea, anæsthesia of the remaining portions of the cornea continued. Hancock's operation—division of the ciliary muscle—relieved the anæsthesia and caused the ulcer of the cornea to heal. This proved, in the opinion of the writer, that the benefits of the operation resulted from the division of the ciliary muscle and not simply from paracentesis of the corneæ. The writer considered it of very great importance, after this operation, to instil frequently upon the conjunctiva a strong solution of atropine, in order to cause the divided fibres of the ciliary muscle to separate and thus prevent their rapid reunion.

### SANITARY MEASURES.

At a recent weekly meeting of the Society, an interesting discussion took place, in reference to the "Metropolitan Health Bill," now before the General Assembly, at Springfield. Prof. Davis, chairman of the committee, composed of Drs. Davis, Hamill, and Ross, read an able report in reference to this subject. We shall inform our readers of the results of legislation on the sanitary regulations of Chicago.

